# Assessment of the Effectiveness of Pharmaceutical Advice in Selected Digestive Disorders: Perspectives of Patients and Pharmacists as Part of a Pilot “Minor Ailments” Service

**DOI:** 10.3390/diseases14020059

**Published:** 2026-02-05

**Authors:** Piotr Merks, Urszula Religioni, Régis Vaillancourt, Dariusz Świetlik, Katarzyna Plagens-Rotman, Ewelina Drelich, Mariola Borowska, Piotr Bromber, Justyna Kaźmierczak, Eliza Blicharska, Paweł Piatkiewicz, Aneta Królak-Ulińska, Radosław Sierpiński, Sebastian Sikorski, Zbigniew Doniec

**Affiliations:** 1 Department of Pharmacology and Clinical Pharmacology, Faculty of Medicine, Collegium Medicum, Cardinal Stefan Wyszyński University, 01-938 Warsaw, Poland; piotrmerks@googlemail.com; 2 School of Public Health, Centre of Postgraduate Medical Education, 01-813 Warsaw, Poland; urszula.religioni@gmail.com (U.R.); piotr.bromber@cmkp.edu.pl (P.B.); 3 Faculty of Medicine, Collegium Medicum, Cardinal Stefan Wyszynski University, 01-938 Warsaw, Poland; regisvaillancourt@hotmail.com (R.V.); p.piatkiewicz@uksw.edu.pl (P.P.); 4 The Polish Pharmacy Practice Research Network (PPPRN), 01-938 Warsaw, Poland; drelichewelina@gmail.com (E.D.); justynamichner@gmail.com (J.K.); 5 Division of Biostatistics and Neural Networks, Medical University of Gdańsk, 80-210 Gdańsk, Poland; dswietlik@gumed.edu.pl; 6 Center for Pediatric, Adolescent Gynecology and Sexology, Division of Gynecology, Department of Gynecology, Poznan University of Medical Sciences, 61-701 Poznan, Poland; plagens.rotman@gmail.com; 7 Department of Pathobiochemistry and Interdisciplinary Applications of Ion Chromatography, Medical University of Lublin, 20-400 Lublin, Poland; eliza.blicharska@umlub.edu.pl; 8 Anesthesiology and Intensive Care Unit, Węgrów Regional Hospital, 01-100 Węgrów, Poland; aneta.k.ulinska@hotmail.com; 9 Department of Administrative Law and Local Government, Cardinal Stefan Wyszyński University, 01-938 Warsaw, Poland; r.sierpinski@uksw.edu.pl (R.S.); s.sikorski@uksw.edu.pl (S.S.); 10 Department of Respiratory Pathophysiology, Institute of Tuberculosis and Lung Diseases, 34-700 Rabka-Zdrój, Poland; zdoniec1@gmail.com; 11 Medical Institute, University of Applied Sciences, 34-400 Nowy Targ, Poland

**Keywords:** pharmaceutical advice, community pharmacy, dyspepsia, GSRS, minor ailments, service acceptability

## Abstract

Introduction: Minor digestive ailments are a common reason for individuals to visit pharmacies, and can be efficiently managed through structured pharmaceutical advice. This study aimed to evaluate the effectiveness of advice provided by pharmacists in community pharmacies from the perspectives of both patients and pharmacists. The primary focus of the study was not on assessing the effectiveness of a specific medication, but rather on the pharmaceutical advice provided. Materials and Methods: This prospective multicenter observational study was conducted between January and March 2025 in community pharmacies across Poland among adult patients with dyspepsia without alarm symptoms and included two visits: an initial visit and a follow-up phone call after 7–14 days. Symptom severity across seven domains was assessed using a GSRS-based tool, and data on adherence, treatment regimen, patient satisfaction, and acceptable costs of the two-visit service were collected. Statistical analyses (*p* < 0.05) using both parametric and non-parametric tests were performed on data from 100 participants who completed the study, with cost data serving as a proxy for willingness to pay. Results: Most patients (92.7%) reported symptom improvement, with a median time to relief of 3 days and good treatment adherence. The greatest benefits were observed for abdominal pain and flatulence, and higher baseline symptom severity was consistently associated with greater improvement. Service acceptability was high, and patients’ reported willingness to pay suggests perceived value and potential economic feasibility of the service. Conclusions: Structured pharmaceutical advice for digestive ailments (including triage, education, management plans, and monitoring of effects) led to rapid and clinically significant improvements in most patients. This approach demonstrates high adherence rates and positive acceptability. The stability of effects across different demographic groups, along with a predictable pattern of changes in various domains, supports the expansion of this service and customization of educational messages.

## 1. Introduction

Upper gastrointestinal disorders, such as dyspepsia, epigastric pain, flatulence, and a sensation of fullness, are among the most commonly reported health issues in adults, imposing a significant burden on healthcare systems. According to recent population data, the prevalence of functional dyspepsia, based on Rome IV criteria, is approximately 6.8–8.4% globally, with higher rates observed in women and marked differences between developed and developing countries [1].

For many patients, community pharmacies serve as the first point of contact for addressing minor ailments. An increasing body of evidence suggests that pharmaceutical consultations enhance accessibility to advice, are well-received by patients, and provide quality care comparable to traditional healthcare services while also being more cost-effective. In Poland, the development of pharmaceutical care (including advice about minor ailments) has been extensively described by Merks et al., who highlighted the potential of pharmacies to alleviate the burden on primary healthcare and enhance the quality of patient care [2].

The role of pharmacists in managing dyspepsia includes an initial patient history, identifying any alarming symptoms, providing education, recommending non-pharmacological interventions, selecting appropriate OTC treatment, and, if necessary, referring patients to a physician. Current guidelines from the British Society of Gastroenterology (BSG) organize the diagnostics and treatment processes for dyspepsia into a practical algorithm, while contemporary recommendations from the American College of Gastroenterology (ACG) support a “test and treat” strategy for Helicobacter pylori [3,4].

To evaluate the effects of pharmaceutical advice, it is important to consider the patient’s perspective (the severity and duration of symptoms, satisfaction, understanding and adherence) as well as the parameters of acceptability and security of intervention. The Gastrointestinal Symptom Rating Scale (GSRS) and its variants, which have been validated for the Polish population [5], are utilized to provide a comprehensive multidimensional assessment of gastrointestinal symptoms.

In Poland, the development of pharmaceutical care services is advancing, with recent research highlighting the need to standardize the advice process and enhance cooperation between physicians and pharmacists. While pharmacists express a high level of acceptance for collaborative models, they also identify barriers, such as a lack of awareness among physicians regarding pharmacists’ competencies [6]. Additionally, there is an interest in implementing “pharmacist-only medicine” (POM) categories, which could enhance the role of pharmacies in treating minor ailments [7]. In the European context, pharmaceutical services began to be systematically implemented in the early 2000s, primarily in Anglo-Saxon countries. The most developed models currently operate in the United Kingdom, Ireland, Canada, and selected Western European countries, where pharmacists provide services such as “minor ailments” programs, vaccinations, medication reviews, and “Pharmacy First” services, often financed from public funds. In Poland, the development of pharmaceutical services remains at an early stage—despite the introduction of a legal framework for pharmaceutical care, most services (except for selected services, such as vaccinations) are currently not publicly funded and are provided as paid services in community pharmacies.

From a health system perspective, minor digestive complaints represent high-volume conditions that consume considerable primary care resources, despite often being manageable through self-care and pharmacy-based interventions. Expanding the role of pharmacists in this area may help to reduce unnecessary general practitioner (GP) visits, shorten waiting times, and improve the allocation of limited healthcare resources. At the same time, the economic sustainability of such services depends on their perceived value by patients and payers, including willingness to pay and the feasibility of integrating these services into reimbursement schemes. Therefore, there is a clear need for empirical data not only on clinical outcomes and patient-reported measures, but also on the economic acceptability of structured pharmaceutical advice for minor upper gastrointestinal ailments.

This study aims to assess the effectiveness of pharmaceutical advice for selected digestive disorders in community pharmacies in Poland, taking into account the opinions of both patients and pharmacists. The evaluation will be based on the Gastrointestinal Symptom Rating Scale (GSRS), time until improvement, patient satisfaction with consultations, and adherence to recommended treatments.

## 2. Materials and Methods

### 2.1. Research Project and Centers

This prospective multicenter observational study was conducted between January and March 2025 in community pharmacies across Poland. Recruitment and observations took place in 23 pharmacies that are part of the Polish Pharmacy Practice Research Network (PPPRN). The process involved two visits: an initial visit to the pharmacy (D0) followed by a follow-up after 7–14 days (a phone call made by a pharmacist).

### 2.2. Inclusion Criteria and Recruitment Process

Adult patients (aged 18 and over) with digestive disorders were invited to participate in the study. Patients presenting with alarm symptoms suggestive of serious gastrointestinal disease, individuals under 18 years of age, and those who did not provide informed consent were excluded from the study.

Participation was voluntary and informed consent was obtained from all participants, including consent for a follow-up contact after 7–14 days. Pharmacists carried out the recruitment during their routine interactions with patients experiencing digestive disorders.

### 2.3. Procedure and Intervention (Pharmaceutical Advice)

During the initial visit, a structured patient history was obtained, including demographic characteristics, current gastrointestinal symptoms and their severity, duration of complaints, comorbidities, and previous treatments. Based on this assessment, patients received pharmaceutical counselling comprising education on the possible causes of symptoms, guidance on appropriate use of OTC medicines, non-pharmacological recommendations (e.g., dietary habits, lifestyle modifications), and an individualized management plan. Although hymecromone (Cholestil Max) was included in the study protocol as a possible symptomatic treatment, the primary focus of the intervention was the provision of structured pharmaceutical advice rather than the evaluation of a specific medicinal product.

Patients were provided with a 7-day treatment log to document medication use, dosage, and adherence. Follow-up assessments were conducted by pharmacists via telephone 7–14 days after the initial visit.

### 2.4. Measurement Tools and Variables

1) The severity of gastrointestinal symptoms was assessed using a standardized questionnaire based on the GSRS (Gastrointestinal Symptom Rating Scale), applied in its previously validated form. This instrument evaluates seven key domains:
  - abdominal pain (right hypochondrium),
  - flatulence,
  - early satiety,
  - persistent feeling of fullness,
  - nausea,
  - frequent belching,
  - diarrhea and/or constipation.

During the initial visit, the severity scale was categorized into four levels: 0 = none, 1 = mild, 2 = moderate, and 3 = severe. In the follow-up assessment, changes in each domain were evaluated using the following scale: 1 = exacerbation, 2 = no change, 3 = improvement, and 4 = significant improvement. The GSRS has been previously validated, including for use in similar clinical and observational settings [8].

2) Adherence

Adherence was assessed based on patient self-report and expressed as the percentage of days during which the recommended treatment regimen was followed over the preceding seven days.

3) Medication regimen

Data were collected on the number of doses taken per day and the total duration of medication use (in days).

4) Service acceptability

Service acceptability was evaluated by asking the participants whether they considered the price of the two-visit package (initial consultation and follow-up) to be satisfactory, along with a voluntary overall satisfaction rating.

5) Pharmacists’ assessment

During follow-up, pharmacists provided a general qualitative assessment of symptom changes and documented the content of the follow-up conversation with the patient.

### 2.5. Final Points

Primary: the effectiveness of pharmaceutical advice was evaluated based on changes in the severity of symptoms (improvement or significant improvement vs. no change or exacerbation), specifically within the domains of the GSRS and, if measured, the overall indicator.

Secondary: (a) the time taken to achieve noticeable improvement, (b) adherence represented as a percentage of time, (c) acceptability or usability (opinions on the price of the service), and (d) comparisons vs. previous symptoms (if applicable).

### 2.6. Sample Size

The analysis of effectiveness comprised 100 participants (N = 100). No formal power calculation was performed; the study was pragmatic in nature.

### 2.7. Statistical Analysis

- Quantitative variables were described using means and standard deviations (SD) or medians and interquartile ranges (IQR), while qualitative variables were described using frequencies and percentages.
- Normality of distribution: assessed using the Shapiro–Wilk test; equality of variances: evaluated with Levene’s test (Brown–Forsythe).
- Comparison of two groups: analyzed using the Student’s t-test or Mann–Whitney U test (depending on whether assumptions were met).
- Qualitative variables: analyzed using χ^2^ (with Yate’s correction for small sample sizes) or Fisher’s exact test.
- Dependencies: assessed with Pearson’s or Spearman’s coefficients.
- Significance level: *p* = 0.05.

Analyses were conducted using Statistica software (version 12.0; StatSoft) and Excel.

### 2.8. Ethics and Data Protection

All methods were carried out in accordance with relevant guidelines and regulations, including the Declaration of Helsinki and applicable national and institutional research standards. The study protocol, including recruitment, information/consent procedures, research instruments, and data-protection measures, was reviewed and approved by the Bioethical Committee at Poznan University of Medical Sciences (Resolution No. 667/24, 9 October 2024).

Written informed consent for participation in the study and follow-up contact was obtained from all participants. The data were anonymized prior to analysis, and the reporting was conducted in an aggregated form.

## 3. Results

The analysis comprised 100 patients, of which 69% were females and 31% were males. The mean age of the participants was 51.8 years (±16.2; 95% CI: 48.5–55.0; range: 19–87). The average height was 168.6 cm (±8.5; 95% CI: 166.9–170.3), and the average body weight was 74.8 kg (±15.1; 95% CI: 71.8–77.8). The majority of participants resided in towns (64.6%), followed by rural areas (19.2%), cities with populations of 500,000 or more (11.1%), and medium-sized cities (5.1%) (Table 1).

The most common comorbidities included hypertension (34%), diabetes (13%), rheumatoid arthritis (9%), thyroid disorders (6%), arrhythmia (4%), Parkinson’s disease (3%), and depressive episodes (2%). At the time of entry, moderate or severe symptoms were prevalent: 33.3% experienced pain in the right hypochondrium compared to 28.3% with mild symptoms; 31.3% reported early satiety, while 11.1% had mild symptoms; and 30.6% felt a persistent sensation of fullness, as opposed to 16.3% with mild symptoms.

After providing pharmaceutical advice, which included taking a medical history, education, creating a management plan, selecting symptomatic treatment, and a follow-up after 7–14 days, the GSRS showed improvements in 92.7% of participants, of which 78.1% reported “improvement”, 14.6% noted “significant improvement”, and 7.3% indicated no changes (Table 2).

The average time taken to achieve noticeable improvement was 3.1 ± 1.5 days (median: three days; IRQ 2.0; range: 0–10 days; 95% CI: 2.8–3.4). This indicates a relatively short time to improvement observed after implementing the recommendations. Over the prior seven days, the average adherence to these recommendations was 77.3% ± 24.2% (median: 90%; IRQ 30; range 0–100%; 95% CI: 72.5–82.2). The average dosing regimen consisted of two or three doses per day (54.1% vs. 40.8%, respectively) for approximately six days (average 5.6 ± 1.7; median: 6). This suggests that the majority of patients followed the recommendations closely.

An analysis of the specific domains showed the highest percentages of “improvement” or “significant improvement” in relation to abdominal pain (79.6%; 61.2% + 18.4%) and flatulence (71.4%; 57.1% + 14.3%). Moderate improvements were observed in persistent sensation of fullness (49.5%) and early satiety (41.8%). However, lower improvement percentages were noted for nausea (33.0%) and for diarrhea or constipation (29.6%). There was also one reported case of exacerbation related to “frequent belching” (1.1%) (Table 3).

In the qualitative assessment, the patients positively rated the effects of the treatment (“Good”: 73.8%, “Perfect”: 6.3%) and the alleviation of symptoms (“Good”: 70.4%, “Perfect”: 4.9%).

Regarding the cost of the two visits, 41.3% of the patients stated that an acceptable cost was above PLN 100, while 23.9% found a cost within the range of PLN 81–90 to be acceptable (Table 4). These findings reflect the patients’ perceived value and acceptability of the service and were used as a proxy for willingness to pay.

In the analyses of dependencies, the global result of effectiveness (GSRS) showed no significant correlation with age (R = −0.03; *p* = 0.7526), body weight (R = 0.03; *p* = 0.7817), or place of residence (R = −0.02; *p* = 0.8341). The distribution of the GSRS categories (2/3/4) did not significantly differ between women and men (*p* = 0.3121). However, there were weak but statistically significant correlations at the domain level: “early satiety” correlated positively with body weight (R = 0.24; *p* = 0.0172), and “frequent belching” correlated positively with age (R = 0.22; *p* = 0.0359). Other relationships were not significant. Notably, within all domains, there was a significant relationship between the initial severity of symptoms and subsequent improvement (R = 0.47–0.78; *p* < 0.0001), which suggest that higher baseline symptom severity was associated with greater reported symptom improvement during the follow-up period (Table 5).

The findings of the study indicate that following community pharmacy consultations, the majority of patients reported a rapid and clinically noticeable improvement in digestive symptoms during the observation period, along with high adherence and positive perceptions of the service. The improvements found following intervention remained stable across different demographics, showing no significant differences based on sex, age or place of residence. Rather, the extent of symptom improvement primarily depended on the severity of symptoms during the initial visit to the pharmacy.

## 4. Discussion

In the study conducted in community pharmacies, the use of structured pharmaceutical advice for minor digestive ailments was associated with rapid and clinically significant effects. Overall, 92.7% of the patients reported improvements or significant improvements, with a median of just three days to notice the first effects. The high level of adherence and comfort in utilizing the service further emphasizes its effectiveness. Notably, the success of the interventions appeared to be independent of age, sex, body weight or place of residence. Some weak yet significant correlations were observed in specific domains (e.g., “early satiety” showed a positive correlation with body weight, while “frequent belching” correlated positively with age). These findings suggest that the foundation of the interventions lies in the high-quality nature of advice (organized patient histories, education, development of a treatment plan, and monitoring). This approach works effectively across diverse patient subgroups and aligns with current data supporting the enhanced role of pharmacists in addressing minor ailments. Similar studies have reported a comparable quality of care, reduced costs, and a high level of patient acceptance of pharmaceutical consultations [9,10,11].

Akers et al. made a comparison between pharmacist care for minor ailments and three traditional healthcare services [9]. They found that the clinical outcomes were equally positive, but at a lower cost, highlighting the potential for pharmacist-led care to alleviate pressures on other levels of the healthcare system [9]. Convergent conclusions emerged from an assessment of patient experiences with the General Practice Community Pharmacist Consultation Service (GP CPCS) in England, where patients are referred from general practice to pharmacists. Positive patient experiences were noted, along with the effective “detection” of minor health issues without the need for a physician visit [10]. At the policy level, this approach reinforces the protocol for a comprehensive evaluation of Pharmacy First services, emphasizing not only clinical aspects but also cross-sector collaboration, equitable access, and cost considerations. Importantly, in settings where such services are publicly funded or reimbursed, cost analyses are directly linked to system-level efficiency and access. In contrast, in the present study, the price-related findings reflect the patients’ perceived value and acceptability of a privately paid service rather than a formal assessment of affordability or population-level economic impact. These priorities align with our findings, which include reduced time to achieve improvement, high acceptability, and a willingness to pay [11].

In our study, the quick improvements observed can be attributed to the unique accessibility of pharmacies as “initial points of contact” for patients. Analyses of geographical accessibility indicate that the majority of the population lives within a short distance of pharmacies, promoting early intervention and reducing therapeutic delays [12]. Moreover, the contemporary role of pharmacies in self-care and public health extends beyond simple OTC recommendations; it includes education, triage, monitoring of treatment effects, and the management of specific health indications [13,14]. Data from studies on the “sore throat test and treat” services suggest that using “pharmacists as the first point of contact” can be effective, safe and supportive for rational antibiotic therapy. These findings reinforce the idea that our advice model can be adapted to other areas concerning minor ailments [15]. In the context of Polish society, there is a high level of confidence in pharmacists’ competencies and a willingness to utilize pharmaceutical services (e.g., vaccinations), which supports the promotion of these interventions [16].

Existing literature on gastrointestinal ailments highlights the importance of conducting algorithms in pharmacies. A pre-post intervention study carried out in Spain showed that implementing an algorithm for gastrointestinal conditions in 134 pharmacies improved symptom recovery, quality of life, and patient satisfaction significantly [17]. Our findings, which reported a high percentage of improvement within 7–14 days and rapid response dynamics, align with these results, even though we used a different assessment tool (GSRS in our studies compared to the domains in the Spanish GIS study). The effectiveness of counselling relies heavily on the preparedness of the personnel involved. Seston et al. demonstrated that pharmacists’ readiness for CPCS (specifically in history-taking, documentation and clinical communication) is critical for delivering quality services and care [18]. These findings are consistent with a review by Hindi et al., which suggests a framework for the quality of pharmaceutical services that includes factors such as patient orientation, accessibility, safety, competency, and integration with local healthcare systems. These elements provide a valuable reference for standardizing advice related to minor ailments [19].

The data regarding the frequency and profile of patients seeking assistance for gastrointestinal disorders in pharmacies provide a valuable epidemiological background for our project. A cross-sectional study conducted in 134 pharmacies across Spain revealed that patients often experience overlapping symptoms (epigastric and retrosternal discomfort). This overlap is linked to a greater functional burden and more frequent use of prescription medications. These findings underscore the importance of pharmaceutical assessment and triage for organizing treatment and referring patients to the appropriate care channels [20]. In this context, our observations, particularly the absence of significant demographic differences in effectiveness and notable dependencies in specific areas, suggest that pharmaceutical advice could be a reliable tool for alleviating common symptoms like abdominal pain and flatulence. There are important implications for implementation that should be highlighted. First, the high cost acceptance (with the most common responses falling between PLN 89–90 and PLN 100 or more for two visits) indicates a viable value-based funding model, especially if complemented by uniform quality standards and training sessions. Second, the consistent effects across demographic profiles support the potential for broad scalability, and the observed correlations in specific domains could favor the customization of educational messages. For example, greater emphasis on dietary strategies could be beneficial for patients experiencing excessive satiety. Third, simple tools can enable the effective monitoring of outcomes and the early detection of failures [13,19]. The limitations of our study include the declarative nature of some indicators, such as time until improvement, adherence, as well as the absence of a control group. On the other hand, the strengths of our research lie in the use of standardized symptom assessments, a two-visit protocol with a follow-up after 7–14 days, an analysis of dependencies, and compliance with multidimensional effect indicators like GSRS, various domains, effect assessments, and comfort levels. In the future, we recommend conducting comparative studies with control groups and performing cost-effectiveness analyses within the Polish healthcare system, preferably using real-world models. From a systemic perspective, integrating advice for minor ailments with primary healthcare (including triage and referral pathways), standardizing quality, and implementing digital solutions for collecting patient-reported outcomes (PROs) and electronic patient-reported outcomes (ePROs) are essential. This has been emphasized in the international literature for years [9,10,11,19].

Our findings confirm that standardized pharmaceutical advice for digestive disorders is effective, timely, and well-received by patients. Coupled with the proven accessibility of pharmacies, the “Pharmacy First” initiative, and the growing maturity of quality frameworks, this approach presents a genuine opportunity to alleviate the burden on primary healthcare physicians while enhancing the patient experience, without compromising the quality of care [9,10,11,12,19].

## 5. Limitations of the Study

We are aware that despite our best efforts, the study has limitations. First, some of the indicators analyzed were declarative in nature (e.g., time to improvement, adherence, and cost acceptability), which carries the risk of subjective assessment. The symptom assessment tool used was a GSRS-based version adapted to the pharmacy practice setting, without psychometric revalidation in the study population. Furthermore, the study was observational in nature and did not include a control group (e.g., no intervention or self-care without contact with a pharmacist), which significantly limits the possibility of inferring cause-and-effect relationships. Furthermore, the presented economic aspects reflect patient-reported acceptability of the service and do not constitute a formal cost-effectiveness analysis or a basis for direct systemic extrapolations.

## 6. Conclusions

This pilot study shows that structured pharmaceutical advice delivered in community pharmacies is an effective and acceptable way to manage minor upper gastrointestinal disorders, with 92.7% of patients reporting improvement and a median time to noticeable benefit of three days. High adherence and a simple dosing regimen indicate that the model is feasible in real-world conditions and can be implemented without major organizational changes. Clinically, the greatest benefits were observed for common symptoms such as abdominal pain and flatulence, and the effects were consistent across demographic subgroups, which supports the scalability of this intervention.

From a health economics perspective, the results suggest that such a service has clear potential to generate value at both the patient and system levels. The observed willingness to pay (most patients accepting a price of PLN 81–90 or even PLN 100+ for a two-visit cycle) indicates that structured pharmaceutical advice is perceived as a meaningful benefit and could be incorporated into co-funded or reimbursed “Minor Ailments” schemes. By shifting first contact for minor digestive complaints from general practitioners to pharmacies, this model could reduce avoidable consultations, shorten time to advice, and improve the allocation of limited primary care resources, while using patient-reported outcomes (PROs) as a basis for quality monitoring and, in the future, for value-based payment. Further research should therefore focus on formal cost-effectiveness and budget impact analyses, as well as longer follow-up, to quantify the economic benefits and inform decisions on scaling and funding the service.

## Figures and Tables

**Table 1:** Demographic profile of the participants.

Category	Measurement/Position	Value
Sex	Male	31 (31.0%)
	Female	69 (69.0%)
Place of residence	Rural areas	19 (19.2%)
	Towns	64 (64.6%)
	Medium-sized cities with populations between 100,000 and 500,000	5 (5.1%)
	Cities with populations of 500,000 or more	11 (11.1%)

**Table 2:** Assessment of changes according to the GSRS.

Category	Value
No changes	7 (7.3%)
Improvement	75 (78.1%)
Significant improvement	14 (14.6%)

**Table 3:** Summary of changes in each of the seven domains.

Domain (the Second Visit)	Category of Change	*n* (%)
Abdominal pain (right hypochondrium)	No changes	20 (20.4%)
	Improvement	60 (61.2%)
	Significant improvement	18 (18.4%)
Flatulence	No changes	28 (28.6%)
	Improvement	56 (57.1%)
	Significant improvement	14 (14.3%)
Early satiety	No changes	57 (58.2%)
	Improvement	36 (36.7%)
	Significant improvement	5 (5.1%)
Persistent sensation of fullness	No changes	49 (50.5%)
	Improvement	41 (42.3%)
	Significant improvement	7 (7.2%)
Nausea	No changes	65 (67.0%)
	Improvement	26 (26.8%)
	Significant improvement	6 (6.2%)
Frequent belching	Exacerbation	1 (1.1%)
	No changes	53 (56.4%)
	Improvement	35 (37.2%)
	Significant improvement	5 (5.3%)
Diarrhea and/or constipation	No changes	69 (70.4%)
	Improvement	18 (18.4%)
	Significant improvement	11 (11.2%)

**Table 4:** Patients’ perception of the effects of pharmaceutical advice and the acceptability of the service.

Area	Category/Response	*n* (%)
Effects (compared to previous product)	Very poor	1 (1.3%)
	Poor	4 (5.0%)
	Average	11 (13.8%)
	Good	59 (73.8%)
	Perfect	5 (6.3%)
Alleviation of symptoms	Poor	4 (4.9%)
	Average	16 (19.8%)
	Good	57 (70.4%)
	Perfect	4 (4.9%)
Acceptable costs (two visits) (1 PLN ≈ 0.25 USD and ≈ 0.23 EUR (exchange rate at the time of the study).	PLN 50–60	14 (15.2%)
	PLN 61–70	14 (15.2%)
	PLN 71–80	4 (4.3%)
	PLN 81–90	22 (23.9%)
	Over PLN 100	38 (41.3%)

**Table 5:** Correlation of changes in symptom domains following advice with age, body weight, and place of residence (R, *p*).

Domain (Change)	Predictor	R	*p*
Abdominal pain (right hypochondrium)	Age	−0.15	0.1454
	Body weight	0.08	0.4589
	Place of residence	−0.06	0.5793
Flatulence	Age	0.01	0.8835
	Body weight	−0.02	0.8682
	Place of residence	−0.13	0.2000
Early satiety	Age	0.04	0.6990
	Body weight	0.24	0.0172
	Place of residence	−0.10	0.3458
Persistent sensation of fullness	Age	0.03	0.7790
	Body weight	0.13	0.1899
	Place of residence	−0.14	0.1751
Nausea	Age	0.03	0.7836
	Body weight	0.11	0.2721
	Place of residence	0.16	0.1234
Frequent belching	Age	0.22	0.0359
	Body weight	0.19	0.0663
	Place of residence	−0.19	0.0707
Diarrhea and/or constipation	Age	−0.03	0.7574
	Body weight	−0.07	0.5101
	Place of residence	0.04	0.6771

## Data Availability

Data availability statements are available from the corresponding author.
